# *Taenionemasinensis* sp. n., the first endemic species of *Taenionema* Banks, 1905 (Plecoptera, Taeniopterygidae) from China

**DOI:** 10.3897/BDJ.11.e104618

**Published:** 2023-06-08

**Authors:** Zhi-Teng Chen, Xiao-Han Ye

**Affiliations:** 1 Jiangsu University of Science and Technology, Zhenjiang, China Jiangsu University of Science and Technology Zhenjiang China; 2 Jiuhuabei Avenue, Quzhou, China Jiuhuabei Avenue Quzhou China

**Keywords:** aquatic insects, morphology, new taxa, stonefly, taxonomy

## Abstract

**Background:**

The taeniopterygid genus *Taenionema* Banks, 1905 currently contains 14 species distributed in the Nearctic and the eastern Palearctic Regions. *Taenionemajaponicum* (Okamoto, 1922) is the only species known from the Eastern Hemisphere, specifically in Japan, Korea, Mongolia, Russia and north-eastern China. The authors recently described the larvae of an undetermined *Taenionema* species, which was supposed to represent a second Palaearctic species.

**New information:**

This paper reports the first endemic species of *Taenionema* Banks, 1905, *Taenionemasinensis* sp. n. from China, which also represents the second species of *Taenionema* from the Eastern Hemisphere. Description and illustrations based on male and female adults are provided. The new species is easily distinguished from all congeners by the bilobed abdominal sternum 9 of the male adult. The female adult is characterised by the posteriorly truncate postgenital plate. The male larva is distinguished by the emarginate subgenital plate and hook-shaped paraprocts.

## Introduction

The genus *Taenionema* Banks, 1905 was described as an independent genus, with *Taenionemaanalis* Banks, 1905 as the type species (Banks 1905). Taeniopterygidae has been used as a family since Klapálek (1905). Needham and Claassen (1925) later synonymised *T.analis* with *Taenionemapacificum* (Banks 1900). Illies (1966) confirmed the familial and generic status of Taeniopterygidae and *Taenionema*, respectively. Stanger and Baumann (1993) conducted a comprehensive revision of *Taenionema* focusing on the morphology of adult males and females.

*Taenionema* currently contains 14 species distributed in the Nearctic and the eastern Palearctic Regions, 13 of which are known from North America (Stanger and Baumann 1993, DeWalt et al. 2023). *Taenionemajaponicum* (Okamoto, 1922) is the only species known from the Eastern Hemisphere, specifically in Japan, Korea, Mongolia, Russia and north-eastern China (Okamoto 1922, Stanger and Baumann 1993, Teslenko 2006, Stewart 2009, Chen 2020). Teslenko (2006) reported the occurrence of *T.japonicum* in Jilin Province of north-eastern China. Chen (2020) provided new images of *T.japonicum* adults based on material from Jilin Province.

Chen and Ye (2020) described and provided illustrations of an undetermined *Taenionema* species based on larvae collected from coastal south-eastern China. The discovery of *Taenionema* larvae in a low-elevation coastal area is unusual as the genus is typically found in creeks and small rivers at higher elevations (Stanger and Baumann 1993). The morphology of the larvae described in Chen and Ye (2020) was also unique, but the adult morphology remained unknown due to the low population density of adults and the difficulty of rearing larvae indoors. However, the second author of the paper recently revisited the locality documented in Chen and Ye (2020) and collected both adults and larvae of the undescribed *Taenionema* species. The adult morphology allows for the proposal of a new species based on these specimens.

## Materials and methods

**Figure 1.**
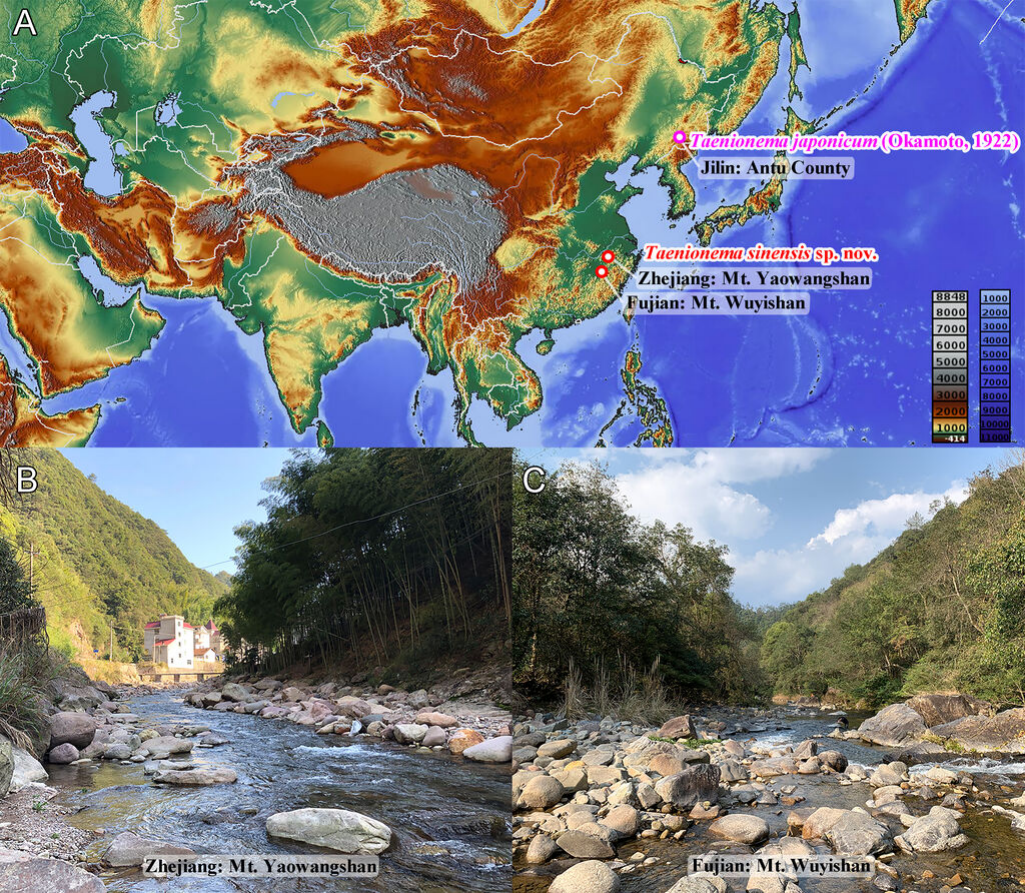
*Taenionema* spp. **A** distribution of *Taenionema* species in China; **B** habitat stream of *Taenionemasinensis* sp. n. in Zhejiang Province; **C** habitat stream of *Taenionemasinensis* sp. n. in Fujian Province.

The adults were collected by sweep-net and light trap in Zhejiang and Fujian Provinces (Fig. 1). The larvae were obtained by dip-net. All specimens used in this study were preserved in 95% ethanol. Observation of external morphology and measurements were performed with an SDPTOP SZM45 stereomicroscope. Photographs were taken with a Canon EOS 6D digital camera, equipped with a Canon MP-E 65 mm 5X macro lens. All images were optimised and assembled in Adobe Photoshop CS6. Type specimens were deposited in the Insect Collection of Jiangsu University of Science and Technology (ICJUST), Jiangsu Province, China. Terminology follows that of Stanger and Baumann (1993).

## Taxon treatments

### Taenionema sinensis sp. n.

CC50A01F-0FE8-5857-A33D-491A548CE817

7D38482C-98A1-4A79-9A25-95ACC116DAF9

#### Materials

- **Type status:** Holotype. **Occurrence:** individualCount: 1; sex: male; **Taxon:** kingdom: Animalia; phylum: Arthropoda; class: Insecta; order: Plecoptera; family: Taeniopterygidae; genus: Taenionema; specificEpithet: *sinensis*; taxonRank: species; nomenclaturalCode: ICZN; **Location:** country: China; stateProvince: Zhejiang; municipality: Quzhou; locality: Yaowangshan; verbatimElevation: 250 m; verbatimCoordinates: 28°46′23″N, 118°58′33″E; **Identification:** identifiedBy: Zhi-Teng Chen; **Event:** verbatimEventDate: 08-03-2020; **Record Level:** institutionCode: ICJUST
- **Type status:** Paratype. **Occurrence:** individualCount: 10; sex: 3 males, 5 females, 2 larvae; occurrenceID: B324F1FF-17C0-5672-863D-A01EE394EC87; **Taxon:** kingdom: Animalia; phylum: Arthropoda; class: Insecta; order: Plecoptera; family: Taeniopterygidae; genus: Taenionema; specificEpithet: *sinensis*; taxonRank: species; nomenclaturalCode: ICZN; **Location:** country: China; stateProvince: Zhejiang; municipality: Quzhou; locality: Yaowangshan; verbatimElevation: 250 m; verbatimCoordinates: 28°46′23″N, 118°58′33″E; **Identification:** identifiedBy: Zhi-Teng Chen; **Event:** verbatimEventDate: 08-03-2020; **Record Level:** institutionCode: ICJUST
- **Type status:** Paratype. **Occurrence:** individualCount: 5; sex: larvae; occurrenceID: A07BC7BA-0984-57F5-AB74-B0646C2CC696; **Taxon:** kingdom: Animalia; phylum: Arthropoda; class: Insecta; order: Plecoptera; family: Taeniopterygidae; genus: Taenionema; specificEpithet: *sinensis*; taxonRank: species; nomenclaturalCode: ICZN; **Location:** country: China; stateProvince: Zhejiang; municipality: Quzhou; locality: Yaowangshan; verbatimElevation: 250 m; verbatimCoordinates: 28°46′23″N, 118°58′33″E; **Identification:** identifiedBy: Zhi-Teng Chen; **Event:** verbatimEventDate: 14-03-2020; **Record Level:** institutionCode: ICJUST
- **Type status:** Paratype. **Occurrence:** individualCount: 11; sex: 1 female, 10 larvae; occurrenceID: 4700AC73-4BE1-5FDD-8B88-CE2D082056B6; **Taxon:** kingdom: Animalia; phylum: Arthropoda; class: Insecta; order: Plecoptera; family: Taeniopterygidae; genus: Taenionema; specificEpithet: *sinensis*; taxonRank: species; nomenclaturalCode: ICZN; **Location:** country: China; stateProvince: Fujian; municipality: Nanping; locality: Wuyishan; verbatimElevation: 730 m; verbatimCoordinates: 27°44′47″N, 117°41′2″E; **Identification:** identifiedBy: Zhi-Teng Chen; **Event:** verbatimEventDate: 08-03-2022; **Record Level:** institutionCode: ICJUST

#### Description

**Figure 2.**
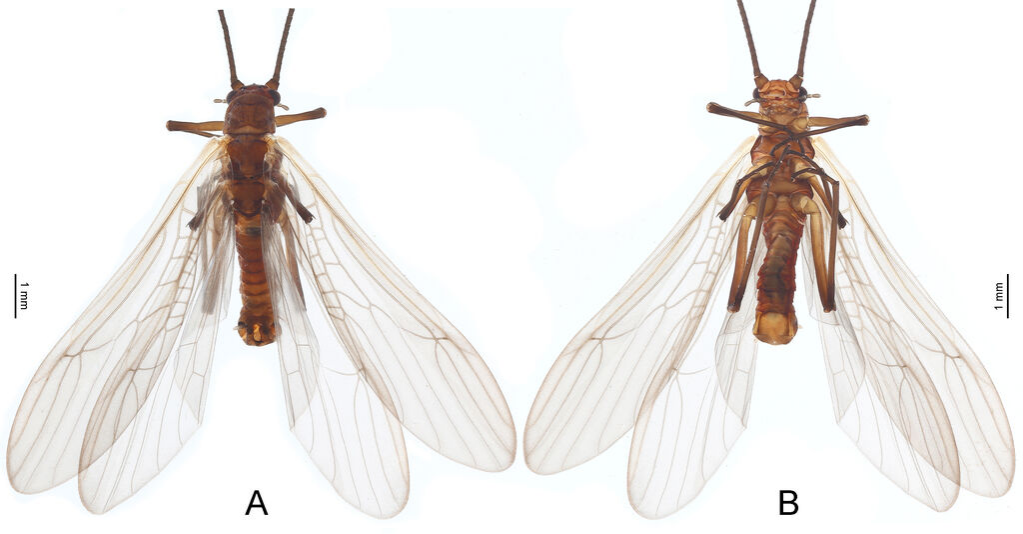
*Taenionemasinensis* sp. n., male holotype, habitus in dorsal view (**A**) and ventral view (**B**).

**Figure 3.**
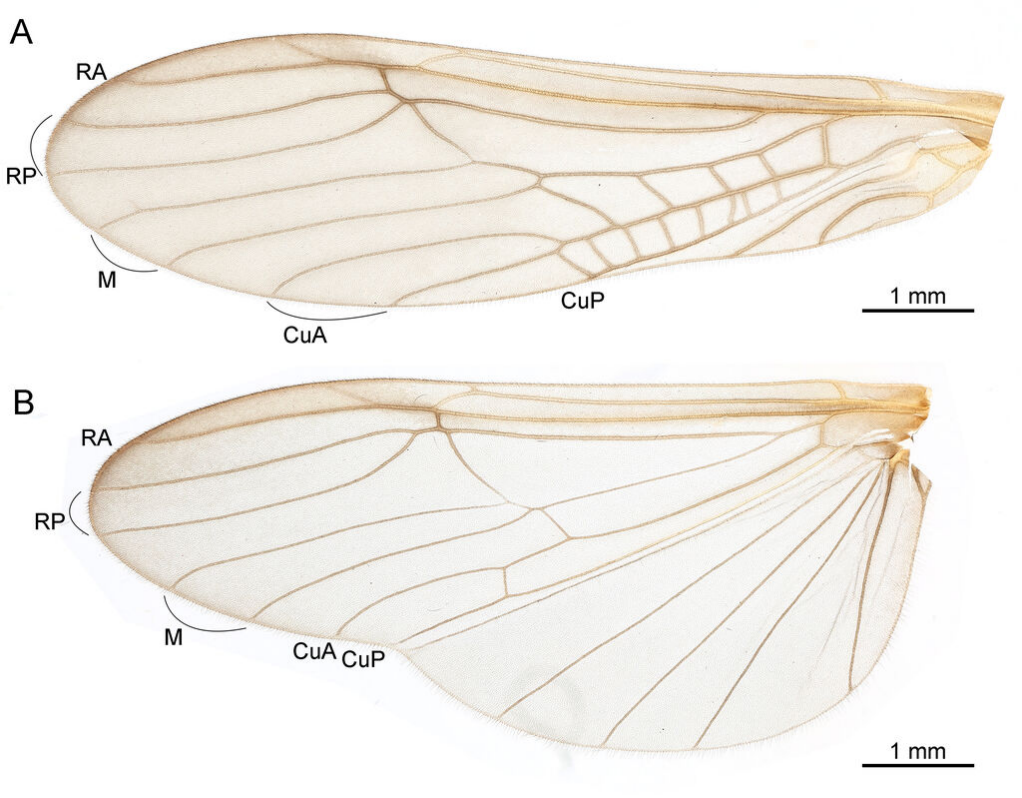
*Taenionemasinensis* sp. n., male holotype, left forewing (**A**) and left hind-wing (**B**). Abbreviations: RA, anterior radius; RP, posterior radius; M, media; CuA, anterior cubitus; CuP, posterior cubitus.

**Figure 4.**
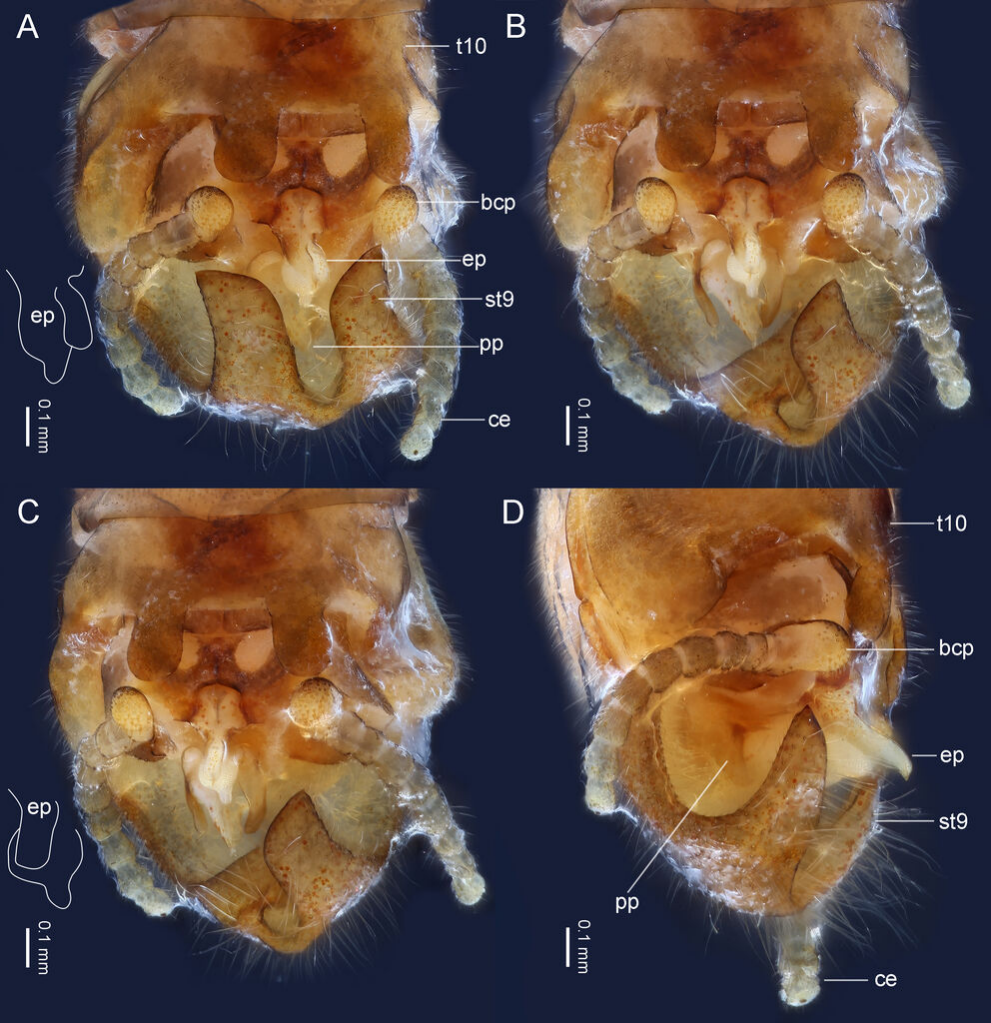
*Taenionemasinensis* sp. n., male holotype, terminalia in dorsal views (**A–C**) and dorsolateral view (**D**). Abbreviations: t10, tergum 10; bcp, basicercal process; ep, epiproct; st9, sternum 9; pp, paraproct; ce, cercus. Shape of epiproct depicted with white lines.

**Figure 5.**
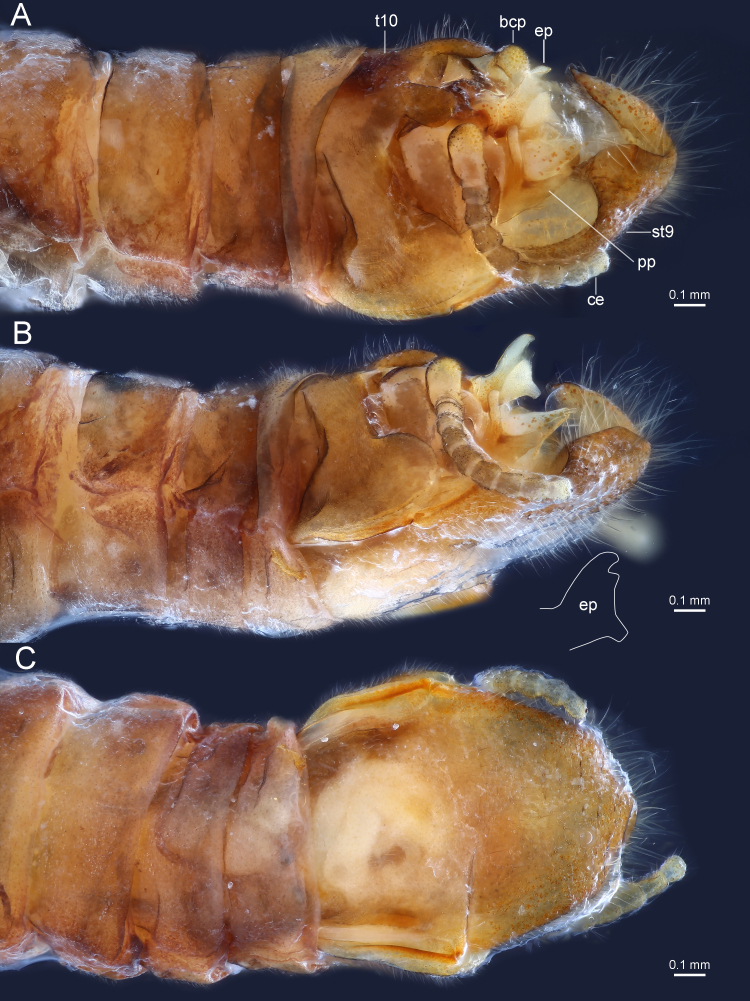
*Taenionemasinensis* sp. n., male holotype, terminalia in dorsolateral view (**A**), lateral view (**B**) and ventral view (**C**). Abbreviations: t10, tergum 10; bcp, basicercal process; ep, epiproct; st9, sternum 9; pp, paraproct; ce, cercus. Shape of epiproct depicted with white lines.

***Male*.** Body length 5.0–7.0 mm (n = 4), colour mostly dark brown (Fig. 2). Head rounded, with three normally-developed ocelli; compound eyes dark. Antennae dark brown and slender, nearly two times longer than body. Pronotum near trapezoidal, bearing scattered rugosities, corners obtuse. Macropterous (Figs 2, 3), forewings length 8.0–8.5 mm (n = 4), hind-wings length 7.0–7.5 mm (n = 4). Wings hyaline, veins brown, without spots. In forewings (Fig. 3), RP with two branches; CuA with two branches. In hind-wings (Fig. 3), RP with two branches; six anal veins present. Legs elongated, brown to dark brown. Abdominal terga 1–9 unmodified. Tergum 10 with two rounded posterior lobes, distance between the lobes equal to the lobe length (Figs 4, 5). Basal plate of tergum 10 sclerotised, lateral struts broad, medial strut broad and forked. Epiproct composed of an upper narrow lobe and a lower bulb, both directed posteriad; the upper lobe expanded laterally and tongue-shaped in dorsal view, finger-shaped in lateral view, gradually tapering to apex; the lower bulb rounded, funnel-shaped in lateral view, posteriorly with a cylindrical projection. Paraprocts asymmetrical, forming three main lobes, including a V-shaped lateral sclerite, a conical median membrane and a finger-shaped lateral sclerite. Cercus eight-segmented, with an ellipsoidal, weakly sclerotised basicercal process, apex with a tiny knob. Sternum 9 broadly-developed, apical half strongly recurved and deeply cleft, forming two pointed lobes.

**Figure 6.**
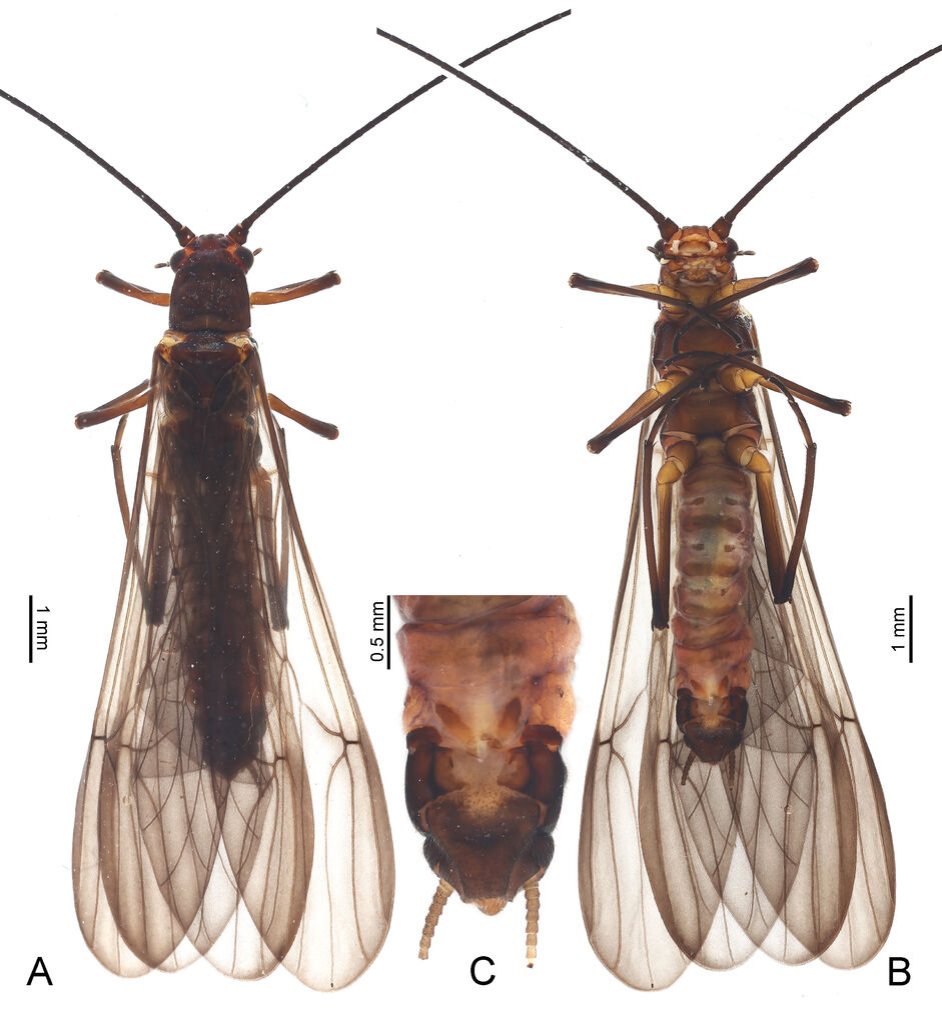
*Taenionemasinensis* sp. n., female paratype **A** habitus in dorsal view; **B** habitus in ventral view; **C** terminalia in ventral view.

***Female.*** Body length 6.0–9.0 mm (n = 5), general colour and pattern similar to the male (Fig. 6). Macropterous; forewing length 9.0–10.0 mm (n = 5), hind-wing length 7.0–8.0 mm (n = 5). The genital opening present on posterior half of sternum 8, with two slender lateral sclerites that are convergent posteriorly (Fig. 6). Cercus six-segmented, apically with a small dark knob. Sternum 9 with two dark longitudinal sclerites lateral to the median membranous area. Postgenital plate broad basally, gradually tapering towards apex, length subequal to basal width, apex truncate or slightly notched.

***Larva.*** Described in Chen and Ye (2020).

#### Diagnosis

*Taenionemasinensis* sp. n. can be distinguished from all congeners by the following characters: adult males with deeply notched, bilobed sternum 9; adult females with a posteriorly truncate postgenital plate; male larvae with a notched subgenital plate and hook-shaped paraprocts.

#### Etymology

The specific epithet refers to the first new species of *Taenionema* known from China, treated as an adjective.

#### Distribution

China: Zhejiang Province; Fujian Province (Fig. 1A).

#### Biology

*Taenionemasinensis* sp. n. has been observed to inhabit moderately-sized creeks (to 10 m wide) characterised by fast-flowing, clear water and boulder and cobble substrates (Fig. 1B and C). Adults of *T.sinensis* sp. n. were primarily found on stream-side plants, with one male adult being captured by a light trap, suggesting a possible positive phototactic response. Adult emergence for *T.sinensis* sp. nov. occurs in early March. Despite the suitable habitat, the population density of *T.sinensis* sp. n. is low in both Zhejiang and Fujian Provinces. The habitat was found to be populated with numerous other stoneflies, including species of Leuctridae, Nemouridae, Peltoperlidae, Perlidae, Perlodidae and Styloperlidae.

## Discussion

Confirmation of conspecificity between adult females and larvae from Fujian Province and those from Zhejiang Province was based on identical female terminalia and especially the larval morphology. This was further supported by the relatively short distance of approximately 170 km between Mt. Yaowangshan in Zhejiang and Mt. Wuyishan in Fujian. The similarity in the unique larval morphology and geographic proximity provides strong evidence for the conspecificity of these specimens.

The classification of the new species within the genus *Taenionema* is supported by several distinguishing characteristics, including forked RP and CuA veins in forewings, a medially depressed and laterally elevated hind margin of male sternum 9, distinct lobes on male tergum 10 and eight-segmented cerci (Ricker and Ross 1975, Teslenko and Zhiltzova 2009). Additionally, the absence of silky cercal fringe hairs in the larvae of *T.sinensis* sp. n., as described in Chen and Ye (2020), is consistent with other species within the *Taenionema* genus (Stewart and Stark 2002, Stewart 2009, Teslenko and Zhiltzova 2009). These morphological features provide strong evidence for the placement of the new species within *Taenionema*.

The newly-discovered species is distinguishable from all of its congeners, including its eastern Palearctic relative, *T.japonicum*, due to several unique features. These include the deeply notched, bilobed sternum 9 in males, posteriorly truncate postgenital plate in females, as well as emarginate subgenital plate and hook-shaped paraprocts in male larvae (Stanger and Baumann 1993, Chen and Ye 2020). These characteristics make the species remarkable and easily identifiable. Stanger and Baumann (1993) have provided a comprehensive key to males and females of *Taenionema*, thus there is no need to provide another key to include the remarkable new species.

*Taenionemajaponicum* is a congeneric species that has been previously known to be widespread in the eastern Palearctic Region, including the Jilin Province of north-eastern China (Stanger and Baumann 1993, Chen 2020). However, the discovery of *T.sinensis* sp. n. in the low-elevation coastal area of south-eastern China has expanded the generic distribution of *Taenionema* in the Eastern Hemisphere in an unexpected way. With this discovery, it is likely that more reports of the genus will surface in the area between Jilin and Zhejiang Provinces.

## Supplementary Material

XML Treatment for Taenionema sinensis
